# MTHFR (methylenetetrahydrofolate reductase: EC 1.5.1.20) SNPs (single-nucleotide polymorphisms) and homocysteine in patients referred for investigation of fertility

**DOI:** 10.1007/s10815-021-02200-6

**Published:** 2021-04-29

**Authors:** Yves Ménézo, Pasquale Patrizio, Silvia Alvarez, Edouard Amar, Michel Brack, Charles Brami, Jacques Chouteau, Arthur Clement, Patrice Clement, Marc Cohen, Dominique Cornet, Brian Dale, Guiseppe D’ Amato, Laetitia Jacquesson-Fournols, Pierre Mares, Paul Neveux, Jean Clement Sage, Edouard Servy, To Minh Huong, Geraldine Viot

**Affiliations:** 1 Laboratoire Clement, Avenue d Eylau, 75016 Paris, France; 2 London Fertility Associates, Harley St, London, UK; 3 Yale University Fertility Center, Orange, CT USA; 4 Clinique de la Muette, 75016 Paris, France; 5 Hopital Américain de Paris, Neuilly-sur-Seine, France; 6 Centre de Santé Ellasanté, 75008 Paris, France; 7 Laboratoire Oriade Noviale, 38000 Grenoble, France; 8 Clinique Natecia, Lyon, France; 9 Centro Fecondazione Assistita, Napoli, Italy; 10 Centro di Fecondazione Assistita di Conversano, Bari, Italy; 11 Centre Hospitalo Universitaire, Nimes, France; 12 Wellmera AG, 4057 Basel, CH Switzerland; 13 VINMEC International Hospital, 458 Minh Khai, Hanoi, Vietnam; 14 IVF Georgia, Augusta, GA USA

**Keywords:** MTHFR SNPs, Homocysteine, Gender prevalence, DNA methylation, Gametes, Fertility, Recurrent miscarriages

## Abstract

**Purpose:**

MTHFR, one of the major enzymes in the folate cycle, is known to acquire single-nucleotide polymorphisms that significantly reduce its activity, resulting in an increase in circulating homocysteine. Methylation processes are of crucial importance in gametogenesis, involved in the regulation of imprinting and epigenetic tags on DNA and histones. We have retrospectively assessed the prevalence of MTHFR SNPs in a population consulting for infertility according to gender and studied the impact of the mutations on circulating homocysteine levels.

**Methods:**

More than 2900 patients having suffered at least two miscarriages (2 to 9) or two failed IVF/ICSI (2 to 10) attempts were included for analysis of MTHFR SNPs C677T and A1298C. Serum homocysteine levels were measured simultaneously.

**Results:**

We observed no difference in the prevalence of different genetic backgrounds between men and women; only 15% of the patients were found to be wild type. More than 40% of the patients are either homozygous for one SNP or compound heterozygous carriers. As expected, the C677T SNP shows the greatest adverse effect on homocysteine accumulation. The impact of MTHFR SNPs on circulating homocysteine is different in men than in women.

**Conclusions:**

Determination of MTHFR SNPs in both men and women must be seriously advocated in the presence of long-standing infertility; male gametes, from MTHFR SNPs carriers, are not exempted from exerting a hazardous impact on fertility. Patients should be informed of the pleiotropic medical implications of these SNPs for their own health, as well as for the health of future children.

## Introduction

**Fig. 1:**
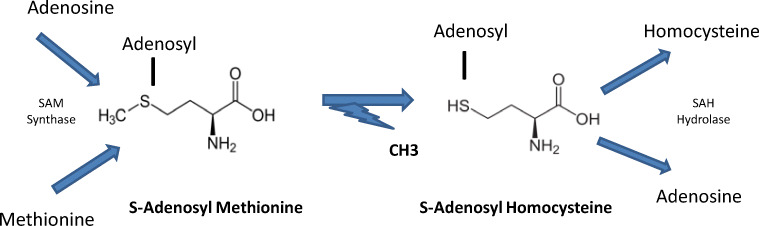
Homocysteine (Hcy) generation. SAH (S-adenosyl homocysteine) is formed after target methylation with release of a methyl group via SAM (S-adenosyl methionine). Free Hcy is then released from SAH by SAH hydrolase

**Fig. 2:**
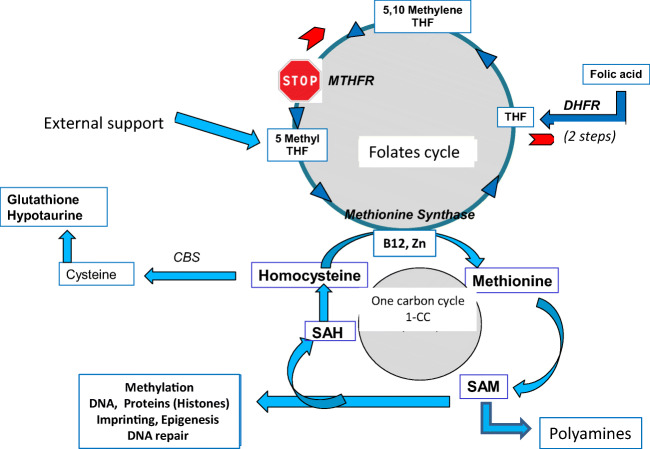
The one-carbon (1-CC) and folate cycles. A narrow gap at the level of MTHFR results in accumulation of unmetabolized folic acid, with feedback inhibition by excess substrate (Michelis and Menten law); the folate cycle may be reversed. A shortage of vitamin B12 (e.g., malabsorption) inactivates methionine synthase, with impairment of methionine synthase activity and accumulation of homocysteine. Mutations in the CBS pathway may also induces Hcy accumulation

It is now generally accepted that human fertility is on a decreasing trajectory, with an increasing time to reach pregnancy (TPP) [1, 2] that is not related to couples’ intent. A drop in sperm quality and male fertility is no longer a matter for debate, and this is widely attributed to environmental factors such as endocrine disruptor chemicals (EDCs) and a variety of pesticides, “the exposome,” known to affect (DNA) methylation process and alter epigenesis [3]. Technical and scientific improvements in assisted reproductive technology (ART) do not provide solutions for all problems related to fertility. EDCs induce oxidative stress, which generates errors in methylation that affect the methylome. Sperm methylome anomalies clearly induce infertility, notably via effects on chromatin structure that interfere with the correct timing of crucial events during preimplantation embryo development. The integrity of the sperm methylome is a major factor necessary for the establishment of full-term pregnancies. The requirement for folate throughout pregnancy and its impact on maternal, fetal, and neonatal health is also widely acknowledged .The process of methylation requires methionine (Met) after adenosylation to S-adenosyl methionine (SAM), the universal methylation cofactor. Target methylation results in formation and release of S-adenosyl homocysteine (SAH) (Fig. 1), which is hydrolyzed to homocysteine (Hcy). Homocysteine is toxic to cells, as it competes with methionine for the same transporter and thus inhibits methylation [4]: it must be regenerated to methionine via the one-carbon cycle (1-CC), supported by the folate cycle. Hcy can also be regenerated via the cystathionine beta synthase pathway (CBS), which is active mainly in the liver but is absent in the human oocyte and early embryo (Fig. 2).

A critical problem in the regeneration of Hcy is linked to the folate cycle: the presence of single-nucleotide polymorphisms (SNPs) that affect the methylene tetrahydrofolate reductase (MTHFR) enzyme prevent formation of 5-MTHF, the active compound, with an optimum yield. This is due to a significant decrease in MTHFR enzymatic activity, particularly in the presence of C677T (Ala222Val) and A1298C (Glu429Ala) SNPs. These mutations have been shown to decrease the metabolic capacity of the enzyme by 70% [5]. Methylation is required for the formation of thymine from uracil; defects may lead to DNA repair and genomic instability [6, 7]. It is a mandatory process in transmission of life: it is a major regulator in gametogenesis, embryo development, and growth via, but only, its role in epigenesis and imprinting mechanisms [8]. Since excess Hcy and the presence of MTHFR SNPs increase pathology risks in general [9–12], the risk of infertility at all stages pre- and post-conception [13–18], the frequency of miscarriage [6, 19, 20], and compromised infant health [21–23], we have determined retrospectively the prevalence of the two main MTHFR SNPs in our population of 2970 male and female patients consulting for infertility; serum homocysteine levels were assessed in parallel.

## Materials and methods

### The population tested

A total of 2970 patients were tested for the two SNPs: 1588 women and 1262 men; they were referred by certified andrologists, gynecologists, or endocrinologists. All of the patients had suffered at least 2 miscarriages (range 2 to 9) and/or at least 2 failed ART attempts (range 2 to 10). The patients were given the option of refusing a blood test for homocysteine assessment. Most of the patients (> 90%) were Caucasian.

### *Biochemical tests*

For the tests, similar protocols were used in all the units in all the units. The testing laboratories are licensed.

*Homocysteine* [24, 25]: fasting blood samples were collected in the morning and serum Hcy measured using the VYTROS kit, which allows determination of homocysteine and homocysteine. Homocysteine is reduced to homocysteine with tris(2-carboxyethyl), and total homocysteine is then transformed into cystathionine in the presence of cystathionine beta synthase (CBS). The cystathionine is hydrolyzed by cystathionine lyase to form Hcy, ammonia, and pyruvate. After reduction with lactic dehydrogenase and NADH to form lactate, the amount of NAD ^+^ produced, proportional to all the homocysteine present in the sample, was measured at 340 nm. The assay is linear from 1 to 90 μmoles/L homocysteine. Cut-off values for dementia and cardiovascular diseases indicate that a level of 10 μmoles/L appears to be a baseline level for healthy patients [10, 11]. A 2.5-μM rise in plasma Hcy concentrations is considered to increase the risk by 10%. We choose a level of 15 μmoles/L as a cut-off for increased risk, and defined three groups according to Hcy concentration: ranges of < 10 moles/L, 10>X<15 and > 15 moles/L

*Genetic testing* [25]: The LAMP human MTHFR mutation kit based on a hybridization technique was used, which requires a 5-μl blood sample. Amplification is performed at 65 °C, using several sets of primers simultaneously. Six specific primers covering the locus of the mutation are used for the 677CT SNP. The same protocol was applied for 1298AC SNP, with 6 specific primers covering the region of the mutation. Two loop primers are used in both, and the probes used simultaneously amplify the wild type gene. The results were evaluated by comparing the curves obtained by fluorescence.

Statistical analysis was carried out by comparing Chi-square percentages.

## Results

Table 1: Gender prevalence of C677T and A1298C MTHFR SNPs. No significant association was observed between sex and the prevalence of mutation type (Chi-square, *p* = 0.056). Mutation was absent in only 16.3 % of the women and 14.6% of the men (15.5% of the total population). Of the population, 21.3% was found to be compound heterozygous C677T/A1298C. Fifty-six percent of the population (55% of the women, 57.5% of the men) was affected (at different degrees) with the 677CT mutation, which is considered to be the most concerning. A few individuals carry 3 mutated alleles (0.4%).

**Table 1:** Distribution of the MTHFR 677CT and 1298AC SNPS. The sex difference in the distribution is not significant (*p* = 0.056). * patients carrying 3 mutations. WT, wild type

Mutation type	Women (%)	Men (%)	Total (%)
677CC/1298AA (WT)	259(16.3)	202(14.6)	461(15.3)
677TT/1298AA	181(11.4)	158(11.4)	339(11.4)
677CC/1298CC	148(9.3)	111(8.)	259(8.7)
677CT/1298AC	308(19.4)	326(23.6)	634(21.3)
677CT/1298AA	381(24)	311(22.5)	692(23.3)
677CC/1298AC	307(19.3)	265(19.2)	572(19.3)
3 allele mutations*	4 (0.0025)	9(0.007)	13(0. 4)

**Fig. 3:**
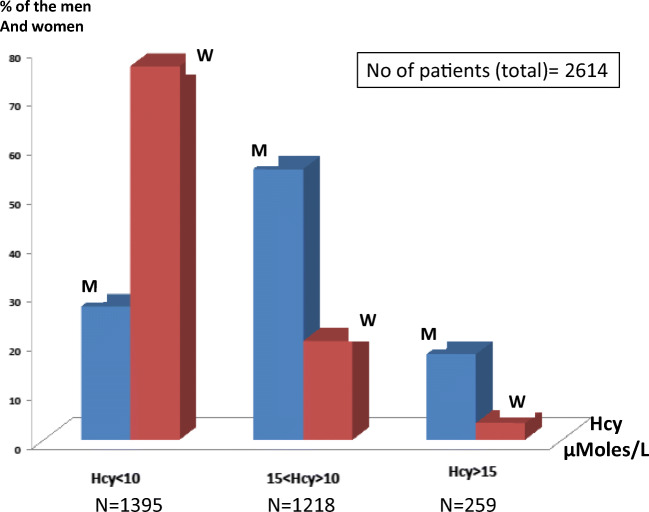
Serum Hcy according to gender (M, men; W, women)

Serum Hcy levels between 3.5 and 110.6 μmoles/L were observed in our overall population. In the total population of 2614 patients tested for Hcy, 1219 patients were found to have Hcy > 10 μmoles/L (46.6% of the patients); 72.8% of the men and 23.5% of the women are included in this range (Fig. 3). Of the men, 17.2 % and only 3.4% of the women had levels over the critical value of 15 μmoles/L (259 patients, 9.9% of the whole population). MTHFR SNPs have a more significant impact on circulating homocysteine levels in men than in women (Chi-square = 594, *p* = 9.8 ^**e-130**^). Thirty one patients had a Hcy >30 μmoles/L (twice the critical value of 15 μmoles/L) 30 men (twenty seven 677TT, one 677CT and two 677CT/1298AC) and one woman (Hcy = 97.3 μmoles/L) carrying no MTHFR mutation.

**Fig. 4:**
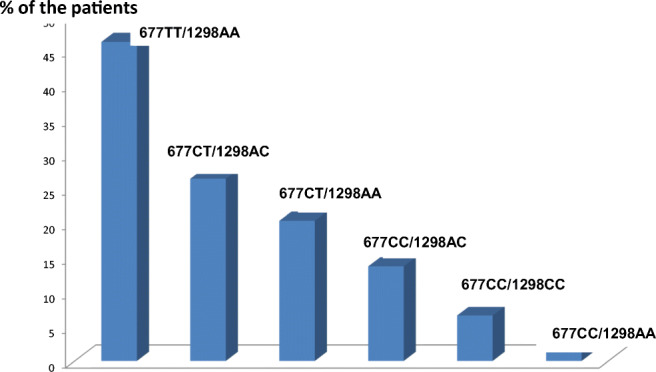
Patients having a blood Hcy > 15 μmoles/L according to the mutational status

Homocysteinemia > 15 μmoles/L and genetic status in the overall population (Fig. 4). As expected, a large majority (more than 2/3) of the elevated homocysteinemia levels were found in individuals carrying the 677CT isoform: 42.6% homozygous (677TT/1298AA) and 26.4% heterozygous composite 677CT/1298AC. If we add to this 20.3% corresponding to the heterozygous 677CT/WT, the 677 isoform is responsible for more than 80% of the cases with elevated Hcy. Two men and one woman carrying a triple allele mutation, 677TT/1298AC had an elevated homocysteine of > 15 μmoles/L. Fourteen patients (0.5%: 12 men and 2 women) had elevated serum Hcy in the absence of an associated MTHFR variant: this can be due to a “folate trap” syndrome or anomalies/mutations in the CBS/MS pathway.

## Discussion and conclusions

Only 15.5% of our population was completely free of C677T and A1298C SNPs MTHFR isoform. More than 50% carry the 677CT SNP at different levels: this mutation is considered to be the most concerning, whatever the health problem. Meta-analyses have already proved the increased risk for carriers of these SNPs to be affected by infertility. In women, these SNPs are considered as a source of “unexplained” infertility [18]. According to Tara et al. [20], men and women carriers of 677TT/WT, 677CT/1298AC, or 1298CC SNPs are most affected. In their study, the SNP distribution and prevalence is by far different from the control group having no problem of conception. In our study, considering their evaluation, 43% of the men and 40 % of the women are in this “at-risk” group as well as 75.6% of the patients with Hcy > 15 μmoles/L. MTHFR isoforms and elevated Hcy levels are negatively associated with the integrity of gametogenesis in both males and females [13–18, 26–35], independently, or not, of the risks of damages affecting the early embryos immediately post conception [23, 26]. In the bovine, MTHFR has been shown to regulate blastocyst development and viability, with an influence on cell number in both the inner cell mass and the trophoblast [27]. This confirms the role of methylation in blastocyst development and fetal development all along pregnancy [8]. Although determination of the 677CT isoform might be sufficient for screening, the 1298AC isoform, also associated with early human developmental anomalies [20, 23, 26] is also well represented. A correct methylome in both gametes is crucial for successful pregnancy [28–35]. A high level of Hcy (97.3 micromolar) was observed in a woman, and to a lesser extent in a marginal number of patients the absence of MTHFR variants: this is probably to the CBS and the MS pathways known to be affected by SNPs . A problem in absorption of vitamin B12 (folate trap) which inhibits methionine synthase activity may also increase Hcy.

Elevated homocysteine is found mainly in the presence of the 677CT isoform: men are significantly more likely to have elevated Hcy than women. This has been previously described in a smaller cohort and attributed to a better capacity for re-methylation in women [36]. The association between MTHFR and elevated homocysteine is obviously different between men and women. However, in the perspective of fertility in general, an active/efficient methylation process is far more important for a gamete genesis process that is continuous, i.e., spermatogenesis, than for one that is not continuous, i.e., oocyte maturation. On a cell and a daily basis, sperm production requires more methyl group than the final steps of maturation of one oocyte. Homocysteine is at the epicenter of oxidative stress and methylation errors [3, 9].

Prior to any ART attempt, dietary supplementation with 5-MTHF (5-methyl tetra hydrofolate: Impryl^R^, Metafolin^R^, Tetrafolic^R^), the compound that lies immediately downstream from MTHFR, should be recommended for both members of the couple, instead of folic acid. This strategy has yielded good results in patients with a lengthy duration of infertility [17]: it decreases serum homocysteine [24, 37], and its safety and efficacy are proven [37–39]. Synthetic folic acid has a low capacity for entering the folate cycle [40, 41] and is very poorly effective and transformed into 5-MTHF in carriers of MTHFR SNPs [28, 29]; Moreover, treatment with folic acid at high doses (5 to 15 mG/day) is known to cause an un-metabolized folic acid (UMFA) accumulation, a source of health questioning [42–47].

Clearly MTHFR SNP determination is not a first-line diagnostic strategy, but it should be recommended for patients with severe infertility of long duration, including repeat miscarriages [17, 19, 20, 23]. A further significant observation merits attention: medical and family histories taken by our geneticists from patients carrying the MTHFR SNPs revealed that some of their relatives suffered cardiovascular or neurological (Alzheimer and other neuropsychiatric) disease. Patients should be informed of these pleiotropic medical implications for their own health, as well as for the health of future children (especially boys).
